# Bat Influenza M2 Shows Functions Similar to Those of Classical Influenza A Viruses

**DOI:** 10.3390/pathogens14060599

**Published:** 2025-06-18

**Authors:** Wenyu Yang, Liping Wang, Lei Shi, Jialin Zhang, Heidi Liu, Jun Wang, Wenjun Ma

**Affiliations:** 1 Department of Veterinary Pathobiology, College of Veterinary Medicine, University of Missouri, Columbia, MO 65211, USA; wyang@fredhutch.org (W.Y.); lwang26@bidmc.harvard.edu (L.W.); lsfd9@missouri.edu (L.S.); jzhang5@fredhutch.org (J.Z.); hhldcb@missouri.edu (H.L.); 2 Department of Molecular Microbiology and Immunology, School of Medicine, University of Missouri, Columbia, MO 65212, USA; 3 MU Center for Influenza and Emerging Infectious Diseases, University of Missouri, Columbia, MO 65211, USA; 4 Department of Medicinal Chemistry, Ernest Mario School of Pharmacy, Rutgers, The State University of New Jersey, Piscataway, NJ 08854, USA; junwang@pharmacy.rutgers.edu

**Keywords:** bat influenza M2, antiviral resistance, ion channel

## Abstract

Novel bat influenza viruses show different features in contrast to classical influenza A viruses (IAVs). The M2 of IAVs functions as an ion channel that plays an important role in virus entry, viral assembly, and release and also serves as the antiviral target. To date, whether bat influenza M2 functions as the ion channel like classical IAV M2 remains unknown. Here, we show that the bat influenza M2 amino acid at position 31 (N/S) is critical for sensitivity to antivirals targeting the ion channel such as amantadine and other tested antivirals and that the amino acids at position 37 (H/G) and 41 (W/A) are crucial for virus replication and survival. The results indicate that bat influenza M2 functions similarly to conventional IAVs despite the low identity between the two.

## 1. Introduction

The influenza A virus (IAV) is an important zoonotic pathogen that can infect birds and mammalian species including humans. IAV infections cause significant morbidity and mortality in humans annually and are responsible for seasonal influenza epidemics and pandemics. According to the antigenicity of the viral surface glycoproteins, hemagglutinin (HA) and neuraminidase (NA), IAVs are divided into H1-H19 and N1-N11 subtypes. The H1-16, H19, and N1-9 subtypes of IAVs have been isolated from waterfowl and shorebirds that are considered the natural hosts of IAVs [1,2,3], while the sequences of the H17-18 and N10-11 subtypes of IAVs were only detected in bats [4,5,6]. Since the genome sequences of H17N10 and H18N11 viruses have been identified from bat samples, the host species of IAVs have been expanded to include bats [4,5,7].

Both H17N10 and H18N11 viruses have similar genomes and are closely related to known IAVs, but they are distinct from conventional IAVs [4,5]. In particular, both surface HA and NA proteins of bat influenza viruses show low identities and different characteristics in contrast to the counterparts of conventional IAVs, resulting in unique features of virus cultivation and cellular receptor usage [8,9,10]. The HA proteins of bat influenza H17N10 and H18N11 viruses do not recognize sialic acid receptors used by conventional IAVs; instead, they employ the MHC class II molecules to mediate cell entry [9,10]. The NA proteins of both H17N10 and H18N11 viruses do not have neuraminidase activity [11], and their exact functions remain unclear [12,13]. The M2 of IAVs is the third membrane protein that plays an important role in virus entry, viral assembly, and release [14,15,16] and is also an antiviral target [17]. The transmembrane domain (amino acids 22-46) of M2 protein is a homotetramer that functions as the proton-selective channel, which causes acidification of the interior of the virus particle to initiate genome transcription and replication [18]. In the M2 pore, a histidine (H) residue at position 37 acts as a proton selectivity filter, and a valine (V) residue at position 27 and a tryptophan (W) residue at position 41 serve as channel gates [19,20,21]. The amantadine family of drug inhibitors targets the ion channel of the M2 protein by binding to 27V and 37H with a high affinity, resulting in physical occlusion and antiviral effects [17,22,23]. Although the amantadine family of antivirals has been shown to be effective and has been widely used for many years, the emergence and persistence of drug-resistant influenza strains have compromised its effectiveness. Mutations in the M2 transmembrane domain including L26F, V27A, A30T/V, S31N, and G34E are known to confer resistance to amantadine [24,25]. In previous studies, the M2-S31N mutation has been shown to be key for amantadine-resistant IAVs, and the M2-S31N mutant persists in 95% of the currently circulating IAVs [26,27]. Furthermore, potentially broadly effective antiviral compounds targeting the M2 ion channel have been developed and were shown to be effective in inhibiting amantadine-resistant IAVs [26,28]. In contrast to other bat influenza internal genes, bat influenza M2 shows high homology with the counterparts of classical IAVs at the nucleotide level but only displays an average 45% identity with those of classical IAVs at the amino acid level. Notably, the M2 of both H17N10 and H18N11 bat influenza viruses has N at position 31, which is associated with adamantane resistance [29].

Wild-type bat H17N10 and H18N11 viruses have been successfully generated using reverse genetics and can replicate in limited cell lines effectively [8]. However, it is still challenging to study these novel viruses since they do not induce cytopathic effects (CPEs) in susceptible cells and require specific conditions to amplify these viruses [8,12]. In our and other former studies, chimeric bat influenza viruses, which contain six internal genes (*PB1*, *PB2*, *PA*, *NP*, *M*, and *NS*) from the bat influenza virus and two surface *HA* and *NA* coding regions from the classical IAVs with respective bat influenza virus gene packaging sequences, replicate efficiently and induce CPEs in normal substrates for classical IAVs [30,31], making them useful tools to study the internal gene functions of bat influenza viruses. In this study, we focus on the characteristics of the bat M2 protein by using the chimeric bat influenza virus to test antiviral compounds including amantadine and compounds that are effective against amandine-resistant influenza strains and by mutating both H37 and W41 amino acids located in the proton channel to determine their impacts on the replication and lifecycle of bat influenza viruses.

## 2. Materials and Methods

### 2.1. Plasmids and Virus Rescue

Six synthesized internal genes (*PB1*, *PB2*, *PA*, *NP*, *M*, and *NS*) from the A/little yellow-shouldered bat/Guatemala/164/2009 (H17N10) virus and two surface gene coding regions (*mHA* and *mNA*) from the PR8 A/Puerto Rico/8/1934 (H1N1) with the respective bat H17N10 gene packaging sequences were cloned into the plasmid PBZ, respectively as described previously [30]. These eight plasmids (pBZ-PB1, -PB2, -PA, -NP, -M, -NS, -mHA, and -mNA) were used to generate the chimeric Bat09:mH1mN1 virus. The *M* gene from the A/flat-faced bat/Peru/033/2010 (H18N11) was cloned into a pHW2000 vector, resulting in the plasmid pHW-H18-M as described previously [30]. To generate a recombinant Bat09:mH1mN1-H18M virus, the plasmid pBZ-M was replaced with the pHW-H18-M in the chimeric Bat09:mH1mN1 genetic background. Single substitutions in H17N10-M2/N31S, H17N10-M2/H37G, H17N10-M2/W41A, H17N10-M2/L36I, H18N11-M2/N31S, H18N11-M2/H37G, or H18N11-M2/W41A and double substitutions in H17N10-M2/L36I W41A were introduced using site-directed mutagenesis PCR based on plasmid pBZ-M or pHW-H18-M, resulting in plasmids with the expected single or double substitutions. To rescue the single- or double-mutant viruses, the plasmid pBZ-M was replaced by the plasmid containing each single or double substitutions in the chimeric Bat09:mH1mN1 genetic background.

To rescue chimeric and recombinant viruses as well as their single- or double-mutant viruses, eight gene plasmids (0.5 μg of each plasmid) were mixed and incubated with 8 μL Lipofectamine^TM^ 3000 (ThermoFisher, Waltham, MA, USA) in 250 μL Opti-MEM medium (ThermoFisher, Waltham, MA, USA) at room temperature for 15 min. The transfection mixture was transferred to 90% confluent 293T cells in 6-well plates and incubated at 37 °C with 5% CO_2_ for 48 h. The 0.5 mL supernatants were then collected and passaged on Madin-Darby canine kidney (MDCK) cells three times in 1.5 mL Minimum Essential Medium (MEM, Gibco, Waltham, MA, USA) with 0.3% bovine serum albumin (BSA, Sigma, St. Louis, MO, USA), 1% antibiotic–antimycotic solution (Gibco, Waltham, MA, USA) and 1 μg/mL TPCK-trypsin (Worthington-Biochem, Lakewood, NJ, USA) in 6-well plates until CPEs were present. The virus titers of each rescued virus were determined by plaque assay on MDCK cells.

### 2.2. Antiviral Compounds

Amantadine was purchased from Sigma (St. Louis, MA, USA), and Jun7-12-2 and Jun7-9 were synthesized and prepared by Dr. Jun Wang’s lab [32,33]. Jun7-12-2 is an M2-S31N inhibitor, and Jun7-9 is a dual inhibitor of M2-WT and M2-S31N.

### 2.3. Virus Growth Kinetics

MDCK cells in 12-well plates were infected with each virus at a multiplicity of infection (MOI) of 0.001. After 1 h of incubation at 37 °C, the supernatants were removed from the infected cells that were then washed twice with phosphate buffered saline (PBS). The MDCK cells were cultured in MEM medium with 0.3% BSA and 1 μg/mL TPCK-trypsin. The supernatants were collected at 12 h, 24 h, 36 h, 48 h, 60 h, and 72 h postinfection, and the virus titer in the supernatants was determined on MDCK cells by plaque assay.

### 2.4. Plaque Assay

To determine virus titers, the supernatant collected from infected cells or virus stock was serially diluted from 10^−1^ to 10^−8^ using MEM infection medium, which contains 0.3% BSA, 1% antibiotic–antimycotic solution, and 1 µg/mL of TPCK-trypsin. Confluent MDCK cells in 6-well plates were infected with the diluted virus and incubated for 1 h at 37 °C, after which the inoculum was removed. Next, 2 mL of MEM agarose overlay, which contains a 1:1 ratio of 2% low-melting agarose (Lonza, Walkersville, MD, USA) and 2 × MEM supplemented with 2 µg/mL of TPCK-treated trypsin, was added to each well. After the agarose solidified at room temperature, the plates were incubated upside down at 37 °C for 2–3 days until translucent plaques appeared. Cold methanol was added into the plate to fix the cells, followed by the crystal violet staining. The number of plaques was counted to determine virus titers using the Reed–Muench method.

To determine the efficacy of antivirals in inhibiting each tested virus, plaque reduction assays were performed. Confluent monolayers of MDCK cells in 6-well plates were infected with approximately 100 PFU/well of each tested virus in MEM medium with 0.3% BSA, 1% antibiotic–antimycotic solution, and 1 μg/mL TPCK-trypsin at 37 °C for 1 h. The inoculum was removed from the infected MDCK cells, which were then washed twice with PBS. The infected cells were treated with different concentrations (30 μM, 10 μM, 3 μM, 1 μM, 0.3 μM, and 0.1 μM) of each antiviral drug (amantadine, Jun7-9, or Jun7-12-2) in 2 mL of MEM agarose overlay per well. After the agarose in each well solidified at room temperature, the plates were incubated upside down at 37 °C for 2–3 days until translucent plaques appeared. The plaque size was measured using ImageJ v1.54g software [34]. The area of each well of the 6-well plate was individually measured and determined to be 9.5 cm^2^. For each indicated virus, the areas of all the plaques with clear boundaries were measured and normalized to 9.5 cm^2^ (shown in mm^2^ format).

### 2.5. Statistical Analysis

The significance of differences between groups and virus titers was analyzed by using one-way ANOVA followed by Tukey’s multiple comparison, two-way ANOVA in GraphPad Prism 10 software (GraphPad Software Inc., San Diego, CA, USA), or Student’s *t* test. All the data are representative of at least three independent experiments, and the values are indicated as mean ± SD. The value *p* < 0.05 was determined to be statistically significant (* *p* < 0.05, ** *p* < 0.01, *** *p* < 0.001, **** *p* < 0.0001).

### 2.6. Data Availability

The data presented in this study are available upon request. Materials are available upon request through the filing of material transfer agreements.

## 3. Results

### 3.1. Generation and Characterization of Chimeric Bat Influenza Virus and Its Recombinant and Single-M2-N31S-Mutant Viruses

**Figure 1:**
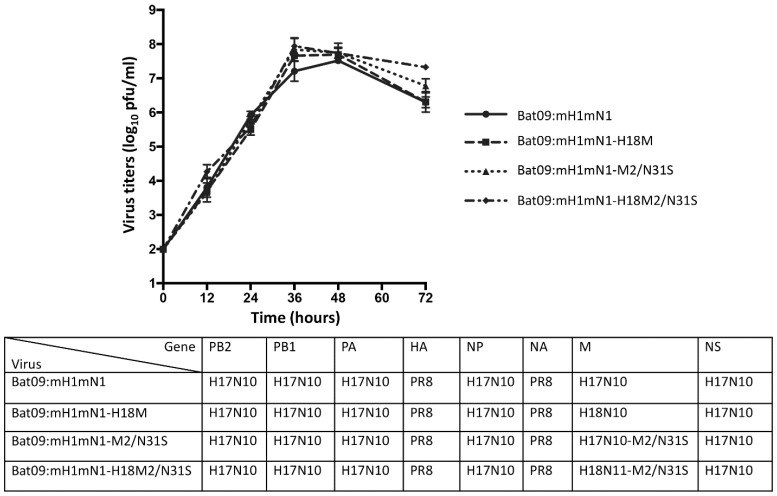
Growth curves of bat influenza recombinant viruses and their M2/N31S mutant viruses. Generated recombinant viruses (Bat09:mH1mN1 and Bat09:mH1mN1-18M) and their M2/N31S mutant viruses (Bat09:mH1mN1-M2/N31S and Bat09:mH1mN1-18M2/N31S), which contain surface gene ORFs from PR8 A/Puerto Rico/8/1934 (H1N1), 5 internal genes from H17N10, and the M gene from either H17N10 or H18N11, were rescued. MDCK cells were infected with each indicated virus at an MOI of 0.001 in 12-well plates. The supernatant was collected at 12, 24, 36, 48, and 72 h postinfection and titrated on MDCK cells by plaque assay. Virus titers are presented as mean ± SD. Their growth curves were determined and compared in three independent experiments and analyzed by two-way ANOVA in GraphPad PRISM 10 software.

As wild-type bat influenza viruses do not cause CPEs in susceptible cells such as MDCK II and RIE1495 [8], the chimeric Bat09:mH1mN1 virus, which has six internal genes from the bat H17N10 virus and two surface HA and NA coding regions from the PR8 H1N1 virus with respective bat H17N10 gene packing signals, was used in this study [30]. To investigate whether M genes from both H17N10 and H18N11 have similar functions and impacts on virus replication, the recombinant Bat09:mH1mN1-H18M was generated using reverse genetics by replacing the M gene of H17N10 with the H18N11 M gene. Previous studies have shown that conventional IAVs with serine (S) at position 31 in M2 are sensitive to amantadine [35,36], while the M2 from either H17N10 or H18N11 virus has an asparagine (N) at position 31, which is expected to confer resistance to amantadine. We generated single-M2-N31S-mutant viruses in the chimeric Bat09:mH1mN1 and recombinant Bat09:mH1mN1-H18M genetic backgrounds, resulting in single-mutant Bat09:mH1mN1-M2-N31S and Bat09:mH1mN1-H18M2-N31S viruses. Growth kinetics analysis showed that both Bat09:mH1mN1 and recombinant Bat09:mH1mN1-H18M replicated to a similar level on MDCK cells (Figure 1). Single N31S substitutions in H17 and H18 M2 in each virus did not impact virus replication on MDCK cells based on growth dynamics when compared with their respective parental viruses (Figure 1).

### 3.2. Chimeric and Recombinant Bat Influenza Viruses as Well as Their Single-Mutant Viruses Show Different Sensitivities to Antivirals Targeting the M2 Ion Channel

**Figure 2:**
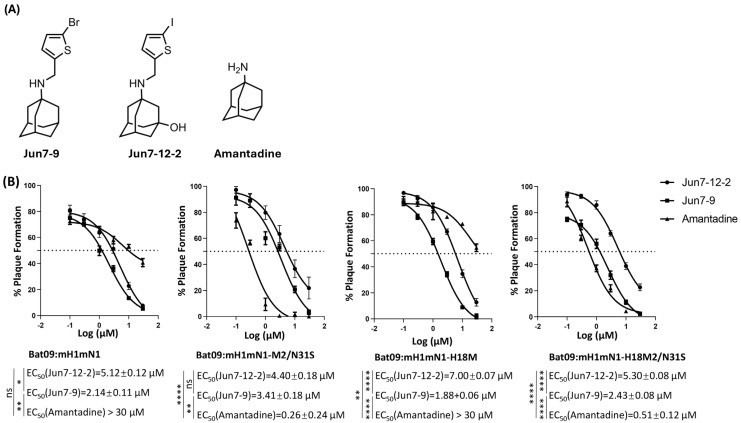
Sensitivity of bat influenza recombinant viruses and their M2/N31S mutant viruses to antivirals targeting the M2 ion channel. Amantadine and compounds Jun7-12-2 and Jun7-9 were used to test the antiviral sensitivity of bat influenza recombinant viruses and their M2/N31S mutant viruses. (**A**) Structures of Jun7-12-2, Jun7-9, and amantadine. (**B**) Sensitivity of bat influenza recombinant viruses and their M2/N31S mutant viruses to the tested antivirals. Each virus was assayed for plaque formation on MDCK cells at different concentrations (30 μM, 10 μM, 3 μM, 1 μM, 0.3 μM, and 0.1 μM) of each tested antiviral. The EC_50_ of each antiviral against each virus was determined by a plaque reduction assay through three independent replicates. The dashed lines in these panels indicate 50% plaque formation. The inhibition efficacy of each compound was analyzed by nonlinear regression (curve fit) in GraphPad PRISM 10 software (ns, not significant; * *p* < 0.05; ** *p* < 0.01; **** *p* < 0.0001).

We determined whether the chimeric and recombinant bat influenza viruses as well as their single-mutant viruses were sensitive to antivirals targeting the M2 ion channel including amantadine, Jun7-12-2, and Jun7-9. Amantadine is an M2-WT (S31) inhibitor and does not inhibit M2-S31N [37]. Jun7-12-2 is an M2-S31N inhibitor and does not inhibit M2-WT (S31) [33]. Jun7-9 is a dual inhibitor of M2-S31N and M2-WT (S31) [32]. Their structures are shown in Figure 2A. The results showed that both chimeric Bat09:mH1mN1 and recombinant Bat09:mH1mN1-H18M were resistant to amantadine as expected, since both M2 from H17N10 and H18N11 have 31N. The antiviral EC_50_ values of amantadine in inhibiting chimeric Bat09:mH1mN1 and recombinant Bat09:mH1mN1-H18M were >30 μM (Figure 2B). In contrast, both Jun7-12-2 and Jun7-9 were more effective in inhibiting both chimeric Bat09:mH1mN1 and recombinant Bat09:mH1mN1-H18M. The antiviral EC_50_ values of Jun7-12-2 in inhibiting chimeric Bat09:mH1mN1 and recombinant Bat09:mH1mN1-H18M were 5.12 ± 0.12 μM and 7.00 ± 0.07 μM, respectively. Jun7-9 showed more-potent antiviral activity against both viruses with EC_50_ values of 2.14 ± 0.11 μM and 1.88 ± 0.06 μM, respectively (Figure 2B).

Both chimeric Bat09:mH1mN1-M2/N31S and recombinant Bat09:mH1mN1-H18M2/N31S with a single N31S mutation in M2 showed high sensitivity to amantadine with EC_50_ values of 0.26 ± 0.24 μM and 0.51 ± 0.12 μM, respectively. Similar to amantadine, Jun7-9 showed potent inhibition against Bat09:mH1mN1-M2/N31S and Bat09:mH1mN1-H18M2/N31S with EC_50_ values of 3.41 ± 0.18 µM and 2.43 ± 0.08 µM, respectively. In contrast, Jun7-12-2 was less effective against both viruses with a single N31S mutation in M2, and no complete inhibition was observed at the highest drug concentration tested (30 µM) (Figure 2B).

In conclusion, chimeric bat influenza viruses with H17N10 or H18N11 M2 carrying N31 are resistant to amantadine but sensitive to both Jun7-12-2 and Jun7-9. Jun7-9 shows inhibition against both amantadine-sensitive and -resistant influenza viruses, while Jun7-12-2 is only active against amantadine-resistant S31N-containing strains.

### 3.3. M2-H37G and M2-W41A Substitutions Critical for the Chimeric H17 but Not for the Recombinant H18 Virus

Both histidine (H) at position 37 and tryptophan (W) at position 41 are the two most conserved residues in the M2 protein of IAVs and are essential for ion channel function [38]. A previous study using a mutagenesis approach indicates that W41 functions as the gate in cooperation with H37 [20]. In contrast to H37, the two mutants (W41A and W41F) that abolish gating [20] actually have enhanced tetramer stability [39], indicating that this residue has evolved for function rather than stability.

**Figure 3:**
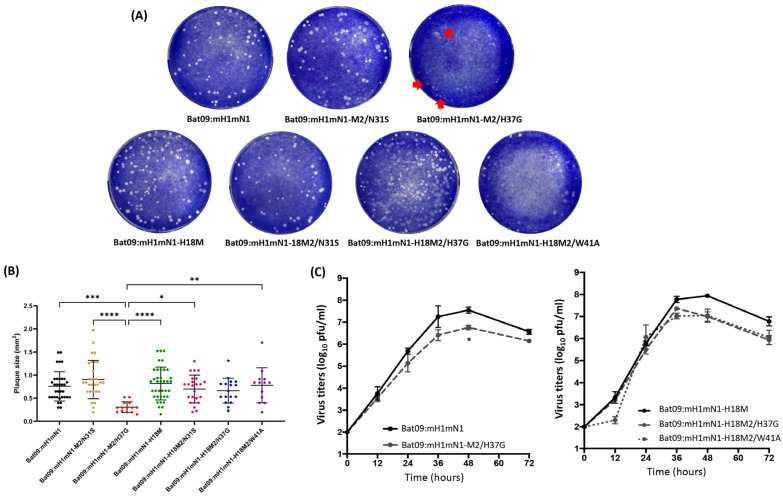
Plaque size and growth curves of bat influenza recombinant viruses and their mutant viruses. (**A**) Plaques formed by bat influenza recombinant viruses and their M2/N31S, M2/H37G, and M2/W41A mutant viruses. The red arrows indicate tiny plaques. The plaque picture represents one of two biological replicates. (**B**) The plaque size formed by each virus was analyzed using ImageJ software. The plaque sizes of each indicated virus were analyzed by one-way ANOVA with follow-up Tukey’s multiple comparison in GraphPad PRISM 10 software (* *p* < 0.05; ** *p* < 0.01; *** *p* < 0.001; **** *p* < 0.0001). (**C**) Growth curves of bat influenza recombinant viruses and their M2/H37G and M2/W41A mutant viruses. MDCK cells were infected with each indicated virus at an MOI of 0.001 in 12-well plates. The supernatants of infected cells were collected at 12, 24, 36, 48, and 72 h postinfection and titrated by plaque assay. Their growth curves were determined and compared across three independent experiments. Error bars show the standard deviation. The growth curves of viruses were analyzed by two-way ANOVA in GraphPad PRISM 10 software (* *p* < 0.05).

To determine the roles of both H37 and W41 in bat influenza M2, we introduced the individual substitutions H37G or W41A in both H17 and H18 M2 and tried to rescue the single-mutant viruses. The results showed that both H18-M2 single-mutant viruses (Bat09:mH1mN1-H18M2/H37G and Bat09:mH1mN1-H18M2/W41A) were successfully rescued, but only the H17-M2/H37G (Bat09:mH1mN1-M2/H37G) single-mutant virus, not the single-mutant virus with W41A in H17-M2, was rescued (Figure 3). Compared with the parental Bat09:mH1mN1 virus, Bat09:mH1mN1-M2/H37G formed significantly smaller plaques on MDCK cells, while the plaque size of the H18-M2 single-mutant viruses was similar to that of those formed by the parental Bat09:mH1mN1-H18M2 virus (Figure 3). The results of growth dynamics revealed that the single H37G mutation in H17-M2 resulted in virus attenuation compared with the parental Bat09:mH1mN1 virus, and significantly lower titers were observed at 48 h postinfection but not at other time points. In contrast, lower titers were found in both Bat09:mH1mN1-H18M2/H37G and Bat09:mH1mN1-H18M2/W41A viruses after 36 h postinfection compared with the parental Bat09:mH1mN1-H18M2 virus, but no significant differences in virus titers were observed among these viruses (Figure 3). All these results indicate that both H37G and W41A substitutions in M2 significantly affect the survival or replication of chimeric viruses in the H17N10 background but do not have a significant impact on chimeric viruses with H18N11-M2.

## 4. Discussion

The identification of novel bat influenza viruses has challenged the dogma that all known IAVs originate from aquatic waterfowl, which are considered the natural reservoir of IAVs. Although significant progress on novel bat influenza viruses has been made [6,40], many questions about these mysterious viruses remain unresolved, such as those regarding NA and M2 functions. In the present study, we determined the M2 characteristics of bat influenza viruses and showed that chimeric bat influenza viruses are resistant to amantadine but sensitive to Jun7-12-2 and Jun7-9, which is consistent with the bat influenza M2 having N31 as expected. When N31 is substituted with S in bat M2, this results in chimeric viruses that are sensitive to amantadine, similar to the findings in conventional IAVs [41,42,43,44,45]. This indicates that the M2 proton channel of bat influenza viruses is a target of the amantadine family of antiviral drugs, similar to that of conventional IAVs.

We further revealed that both His 37 as a proton-selective filter and Trp 41 as a channel gate in the M2 proton channel from H17N10 and H18N11 viruses show different impacts on virus survival and replication. Both H37G and W41A substitutions in H18 M2 do not impact virus survival since single-mutant viruses with either substitution can be rescued. However, they still affect virus replication (lower virus titers were detected at 48 h and 72 h) in contrast to the parental Bat09:mH1mN1-H18M2 virus, despite similar plaque sizes being observed among single-mutant and parental Bat09:mH1mN1-H18M2 viruses. Interestingly, both single-mutant viruses (Bat09:mH1mN1-H18M2/H37G and Bat09:mH1mN1-H18M2/W41A) show similar growth dynamics on MDCK cells. In contrast, the single W41A substitution in H17 M2 results in failure to rescue the virus, while the single H37G substitution does not impact virus survival but causes the formation of small plaques in the single-mutant Bat09:mH1mN1-M/H37G virus when compared with the parental virus. The M2 transmembrane domain between amino acids 22 and 46 in H17N10 and H18N11 viruses is very conserved; there is only one amino acid difference at position 36 between the two M2 proteins (L36 in H17-M2; I36 in H18-M2). Our results showed that the single-mutant virus with the L36I substitution (H17N10-M2/L36I) or the double-mutant virus with L36I and W41A (H17N10-M2/L36I W41A) in H17-M2 cannot be rescued. In contrast, these two amino acids in the M2 ion channel do not impact H18N11 M2 functions in the chimeric viruses. These results suggest that the loss of the M2 ion channel gate function is fatal for the H17N10 M2, and impaired functioning of the M2 proton selectivity filter might affect the replication of the H17N10 M2-based viruses. The difference in rescuing the single-W41A-mutant virus in both H17 and H18 M2 could also be due to the genetic background used, which may impact protein–protein interactions, as our previous studies have shown that uncoating of the recombinant PR8 H1N1 viruses driven by the H17N10 M2 ion channel is much less efficient than that driven by the H18N11 M2 [46]. The underlying mechanisms by which both bat M2s show different features remain unknown and need to be investigated in future studies.

Although M2 proton channel activity is necessary for virus replication and the viral lifecycle, some IAVs are able to survive and replicate without proton channel activity. In a previous study, Watanabe et al. found that the A/Udorn/307/72 (H3N2) virus can undergo multiple cycles of replication without M2 proton channel activity due to the deletion of M2 amino acids 29-31 [47]. It should be noted that the heart of acid activation and proton conductance is H_37_xxxW_41_ in the M2 transmembrane region [20]. The H37G substitution in the M2 proton channel has been confirmed as a poorly selective cation channel [19], and the W41A substitution in the M2 results in abolished gating [20]. It remains unclear whether and how the mutation of H_37_xxxW_41_ actually affects the virus replication or lifecycle of IAVs since conflicting results have been obtained in previous studies. Gannage et al. showed that a recombinant PR8 H1N1 that contains only the extracellular domain of M2 (amino acids 1-25) is able to replicate for a single round in vitro [48]. Wang et al. failed to rescue the recombinant A/duck/Hubei/Hangmei01/2006 (H5N1) virus that had the single H37G substitution in M2 [49]. Jing et al. successfully rescued the A/Udorn/72 (H3N2) virus with the M2-W41A substitution that replicates very efficiently on MDCK cells [50]. In the present study, we were able to rescue the recombinant virus with either H37G or W41A substitution in H18 M2 but only with H37G and not W41A substitution in H17 M2. According to previous studies and our results, this phenomenon is most likely strain-dependent, and the underlying mechanisms need to be investigated in future studies. Taken together, our results demonstrate that the M2 of novel bat influenza viruses has similar proton channel functions to those of classical IAVs, which impacts virus replication and antiviral resistance.

## Data Availability

The original contributions presented in this study are included in the article. Further inquiries can be directed to the corresponding author.
